# Virtual screening and experimental validation of novel histone deacetylase inhibitors

**DOI:** 10.1186/s40360-016-0075-8

**Published:** 2016-07-21

**Authors:** Yan-xin Huang, Jian Zhao, Qiu-hang Song, Li-hua Zheng, Cong Fan, Ting-ting Liu, Yong-li Bao, Lu-guo Sun, Li-biao Zhang, Yu-xin Li

**Affiliations:** National Engineering Laboratory for Druggable Gene and Protein Screening, Northeast Normal University, Changchun, 130024 China; School of Computer Science and Information Technology, Northeast Normal University, Changchun, 130117 China; Research Center of Agriculture and Medicine Gene Engineering of Ministry of Education, Northeast Normal University, ChangChun, 130117 China

**Keywords:** HDAC inhibitors, Pharmacophore, Docking, Virtual screening, Apoptosis

## Abstract

**Background:**

Histone deacetylases (HDACs) are promising therapeutic targets for the treatment of cancer, diabetes and other human diseases. HDAC inhibitors, as a new class of potential therapeutic agents, have attracted a great deal of interest for both research and clinical applications. Increasing efforts have been focused on the discovery of HDAC inhibitors and some HDAC inhibitors have been approved for use in cancer therapy. However, most HDAC inhibitors, including the clinically approved agents, do not selectively inhibit the deacetylase activity of class I and II HDAC isforms, and many suffer from metabolic instability. This study aims to identify new HDAC inhibitors by using a high-throughput virtual screening approach.

**Methods:**

An integration of in silico virtual screening and *in vitro* experimental validation was used to identify novel HDAC inhibitors from a chemical database.

**Results:**

A virtual screening workflow for HDAC inhibitors were created by integrating ligand- and receptor- based virtual screening methods. Using the virtual screening workflow, 22 hit compounds were selected and further tested via *in vitro* assays. Enzyme inhibition assays showed that three of the 22 compounds had HDAC inhibitory properties. Among these three compounds, ZINC12555961 significantly inhibited HDAC activity. Further *in vitro* experiments indicated that ZINC12555961 can selectively inhibit proliferation and promote apoptosis of cancer cells.

**Conclusions:**

In summary, our study presents three new and potent HDAC inhibitors and one of these HDAC inhibitors shows anti-proliferative and apoptosis-inducing activity against various cancer cell lines. These results suggest that the developed virtual screening workflow can provide a useful source of information for the screening and validation of new HDAC inhibitors. The new-found HDAC inhibitors are worthy to further and more comprehensive investigations.

**Electronic supplementary material:**

The online version of this article (doi:10.1186/s40360-016-0075-8) contains supplementary material, which is available to authorized users.

## Background

The dynamic post-translational modification of nucleosomal histones plays a critical role in transcriptional regulation. Hyperacetylation of nucleosomal core histones results in transcriptional activation, while their hypoacetylation leads to transcriptional repression. Modifications of nucleosomal histone acetylation and deacetylation affect the chromatin structure and related gene expression, and thus regulate various cellular processes, including DNA synthesis, cell division and differentiation, apoptosis, and others [1, 2]. The level of histone acetylation is determined by histone acetyltransferase (HAT) and histone deacetylase (HDAC) activities [3, 4]. Impaired HDAC activity could interfere with the balance between HATs and HDACs and thus alter the transcriptional status of many genes, in particular those related to disease. Therefore, HDACs have become promising therapeutic targets for the treatment of cancer, diabetes, and other human diseases [5, 6]. HDACs are classified into four classes (Classes I–IV) depending on their sequence identity and domain organization. Classes I (HDACs 1–3 and 8), II (HDACs 4–7, 9, and 10), and IV (HDAC 11) are referred to as classical HDACs and are generally simultaneously targeted by most HDAC inhibitors [7]. Class III HDACs include Sirt1–7; they are nicotinamide (NAD)-dependent and are insensitive to HDAC inhibitors [8]. To date, a number of HDAC inhibitors have been reported and they can be divided into several structural categories: hydroxamic acids, aliphatic acids, benzamides, cyclic peptides and others [9–11]. HDAC inhibitors have emerged as a new class of therapeutic agents and have generated much interest among pharmacologists, and cancer and diabetes researchers [5, 12, 13]. Three HDAC inhibitors, vorinostat (SAHA) [14], cyclic peptide FK228 (romidepsin) [15, 16] and belinostat [17], have been approved by the U.S. Food and Drug Administration (FDA) for the treatment of cutaneous and peripheral T cell lymphoma. However, most HDAC inhibitors, including the clinically approved agents, non-selectively inhibit the deacetylase activity of class I and II HDACs, and many suffer from metabolic instability. These characteristics have been associated with reduced potency and toxic side effects *in vivo* [18]. Significant efforts are ongoing to address these and other deficiencies of HDAC inhibitors to improve their HDAC inhibitory potential for the treatment of cancer and other diseases [19–21]. In addition, substantial efforts have been made to develop new HDAC inhibitors with potential therapeutic applications [22]. In the present study, we present a hierarchical virtual screening protocol with SYBYL-*X*2.0 [23] and Gold 5.2 [24] software suites for the identification of compounds as potential HDAC inhibitors. It provides a stable and reliable solution for virtual screening of HDAC inhibitors based on commercial software’s of drug discovery. A pharmacophore model was built and used for virtual screening to identify candidate compounds from the enamine dataset in the ZINC database [25]. Then, the remaining compounds were docked into the active site of HDAC8. Finally, 22 compounds were identified as the final hit compounds. Enzyme inhibition assays with the HDAC inhibitor drug screening kit showed that three of the 22 compounds had HDAC inhibitory properties. Among these three compounds, ZINC12555961 was confirmed to have significant inhibitory activity against HDACs. Further *in vitro* cell experiments demonstrated that ZINC12555961 can selectively inhibit proliferation and promote apoptosis of cancer cells.

## Methods

### Pharmacophore modeling

The GALAHAD module in SYBYL-X 2.0 was adopted for ligand-based pharmacophore modeling. Seven hydroxamic acid inhibitors (marked with * in Table 1) with structural diversity were selected as representative compounds. All parameters were set to their default values (such as aligning molecules with pharmacophore features, no molecular template used, etc.) with the exception of 150 generations and a population size of 100. In the virtual screening process performed with the UNITY module in SYBYL-*X*2.0, at least five out of seven features in the pharmacophore model had to be matched.

**Table 1:** Seven compounds for generating pharmacophore models

Name	Structure	Ref.
BindingDB_50114811		[45, 48]
BindingDB_50114835		[31, 45, 48]
BindingDB_50123975		[44]
BindingDB_50214436		[31]
NK308		[46]
SAHA		[31, 46, 47]
TSA		[31, 47]

### Molecular docking

GOLD 5.2 was adopted for molecular docking screening. HDAC8 (PDB id: 1 T69) was selected as the docking target. All the water molecules in HDAC8 were removed and hydrogen atoms were added. The binding site of HDAC8 was defined as those residues within 10 Å from the ligand in the X-ray structures. The parameters of the genetic algorithm (GA) were used in default values (such as the population size of 100, the selection pressure of 1.1, etc.) except that ligands were subjected to 30 GA runs, the number of operations was set to 300,000, and the early termination option was turned off. The three top scoring conformations of every ligand were retained at the end of the calculation. Two of the fitness functions implemented in GOLD 5.2, ChemPLP and ChemScore were used in our experiments.

### HDAC inhibitory activity assay

The HDAC inhibitor drug screening kit (k340-100, BioVision, CA, USA) was used to measure HDAC inhibitory activities of the candidate compounds according to the manufacturer’s instructions. The candidate compounds, assay buffer, and HDAC fluorometric substrate, which comprises an acetylated lysine side chain, were added to HeLa nuclear extracts in a 96-well plate and incubated at 37 °C for 30 min. The reaction was stopped by adding lysine developer, and the mixture was incubated for another 30 min at 37 °C. An additional positive control included incubation with double-distilled water, and the inhibitor control consisted of incubation with Trichostatin A (TSA) at 20 μM. HDAC activities were quantified by a fluorescence plate reader (POLARstar OPTIMA, BMG, BRD) with excitation at 370 nm and emission at 450 nm.

### Cell lines

Four cell lines, namely HepG2 (human hepatocellular carcinoma cell line), L02 (human normal liver cell line), MDA-MB-231 (human breast cancer cell line), and MCF-10A (human normal breast cell line), were obtained from the Cell Bank of the Chinese Academy of Sciences (Shanghai, China). Cells were cultured in an appropriate medium supplemented with 10 % fetal bovine serum (TBD Science, Tianjin, China), 100U/ml penicillin and 100 mg/mL streptomycin (Ameresco, US) at 37 °C and 5 % CO2.

### MTT assay

The MTT (3-(4, 5-dimethylthia-zol-2-yl)-2, 5-diphenyl tetrazolium bromide) assay was used to examine the effects of the candidate compounds on cell viability. The candidate compounds were dissolved in DMSO (dimethyl sulfoxide) as 10 mM/L stock solutions. Cells were plated in 96-well plates (1 × 10^4^ cells/well) in 100 μL of growth medium and allowed to grow for 24 h. The cells were then treated with 0, 1, 10, 50 and 100 μM of each candidate compound in the presence of 3 % serum. After 44 h of treatment, 20 μL of MTT [5 mg/mL in phosphate-buffered saline (PBS); Sigma Chemical Co.] were added to each well for an additional 4 h of incubation. The blue MTT formazan precipitate was dissolved in 100 μL of DMSO. The optical density of samples was measured at 570 nm using a micro ELISA reader (Bio-Rad, Hercules, CA). Cell viability was expressed as a percentage relative to the untreated control cells.

### DAPI staining assay

A DAPI staining assay was performed to reveal the presence of condensing nuclei and apoptotic bodies in compound-treated cells. HepG2 and MDA-MB-231 cells were treated with the candidate compounds (60 and 90 μM) for 48 h, and then harvested, fixed with 4 % paraformaldehyde for 30 min, washed with PBS, and stained with DAPI at a final concentration of 0.5 μg/mL for 15 min at room temperature. The cells were then analyzed using a fluorescence microscope. Three independent experiments were performed, and at least four different fields with a minimum of 100 cells/field were scored.

### Apoptosis assay

Annexin V-FITC/PI (propidium iodide) assay was performed to evaluate apoptosis of cancer cells induced by the hit compound ZINC12555961. HepG2 and MDA-MB-231 cells were seeded on 6-well plates at a density of 1 × 10^6^ cells/well, and incubated with 90 μM of ZINC12555961 for 48 h. Then, the cells were harvested by trypsinization, washed in ice-cold PBS, and resuspended in 190 μL binding buffer containing 5 μL Annexin V and 10 μL PI (Beyotime, China). The cells were incubated in the dark for 10 min and then analyzed by flow cytometry (BD FACSCanto™).

### Cell cycle analysis

DNA staining with PI (Beyotime, China) was used to determine the cell cycle distribution of compound-treated cells. The number of cells at specific phases of the cell cycle was analyzed and sorted using flow cytometry. HepG2 and MDA-MB-231 cells were seeded at a density of 1 × 10^6^ cells/well. After treatment, the cells were collected, washed with PBS, fixed with 50 % alcohol and stained with PI at a final concentration of 1 mg/mL for 30 min. The percentages of cells in different phases of the cell cycle were measured with a flow cytometer (BD FACSCantoTM) and analyzed with the Modfit software (Verity Software House, Topsham, USA).

## Results

### Pharmacophore-based virtual screening

The Enamine dataset in the ZINC database, containing collection of 1.8 million structurally diverse compounds, were used as the screening compound set [25]. As listed in Table 1, seven compounds were used to generate pharmacophore models. 50 hydroxamic acid-based HDAC inhibitors collected from the literature [26–31] and Enamine_p0.18, a subset of the enamine dataset [25], which contains 22,565 compounds, were combined as the test dataset. Moreover, the maximal unbiased benchmarking data sets for HDACs (MUBD-HDACs) that cover all classical HDACs, composed of 631 HDAC inhibitors and 24,609 unbiased decoys [32], were further used to validate our pharmacophore models. The HDAC inhibitor data sets in MUBD-HDACs have been validated extensively as chemically diverse, while the decoy sets are shown to be property-matching with ligands but have no HDAC inhibitory activities. MUBD-HDACs is freely available at http://www.xswlab.org/. All compounds were minimized under the Tripos Standard (TS) force field with Gasteiger-Hückel atomic partial charges. Minimizations were done using the Powell method and terminated at an energy gradient value of 0.01 kcal/mol. All models derived from at least six ligands of the training set (N_NITS ≥ 6) are listed in Table 2, except those models with high energies. Constructing a good pharmacophore model requires balancing among various criteria such as maximizing steric consensus (STERICS), maximizing the pharmacophore consensus (HBOND), and minimizing strain energy (ENERGY) [33]. SPECIFICITY is a logarithmic indicator of the expected discrimination for each model. It is based on the number of features contained, their distribution across any partial match constraints, and the degree to which the features are separated in space. A good pharmacophore model usually has a higher SPECIFICITY value, a higher steric score and a lower energy value. MODEL_001, MODEL_002, MODEL_005, MODEL_006, MODEL_010, MODEL_021, MODEL_030, and MODEL_041 were selected to further validate their screening abilities for the test dataset and the decoy dataset. The enrichment factor (EF) was calculated using equation (1):

**Table 2:** Pharmacophore models generated by GALAHAD

Name	SPECIFICITY	N_NITS	ENERGY	STERICS	HBOND
MODEL_001	2.762	6	59.04	1807.4	255.3
MODEL_002	2.321	7	44.53	1724.8	240.0
MODEL_005	3.842	6	42.64	1585.6	241.8
MODEL_006	5.225	6	55.97	1822.2	239.9
MODEL_010	2.208	7	48.49	1714.7	243.7
MODEL_021	4.220	6	42.25	1551.0	224.6
MODEL_030	2.821	6	44.05	1610.4	231.5
MODEL_035	1.094	6	40.95	1630.8	235.7
MODEL_037	1.651	6	44.93	1714.6	233.4
MODEL_041	2.545	6	39.68	1521.0	202.5

1 $$ \mathrm{E}\mathrm{F} = \left(Ha\times D\right)\ /\ \left(Ht\times A\right) $$

Where *D* indicates the total number of compounds in the test datasets; *A* means the total number of known inhibitors in the test datasets; *Ht* is the hit number of compounds retrieved from the test datasets; and *Ha* represents the number of known inhibitors in the hit compounds.

**Fig. 1:**
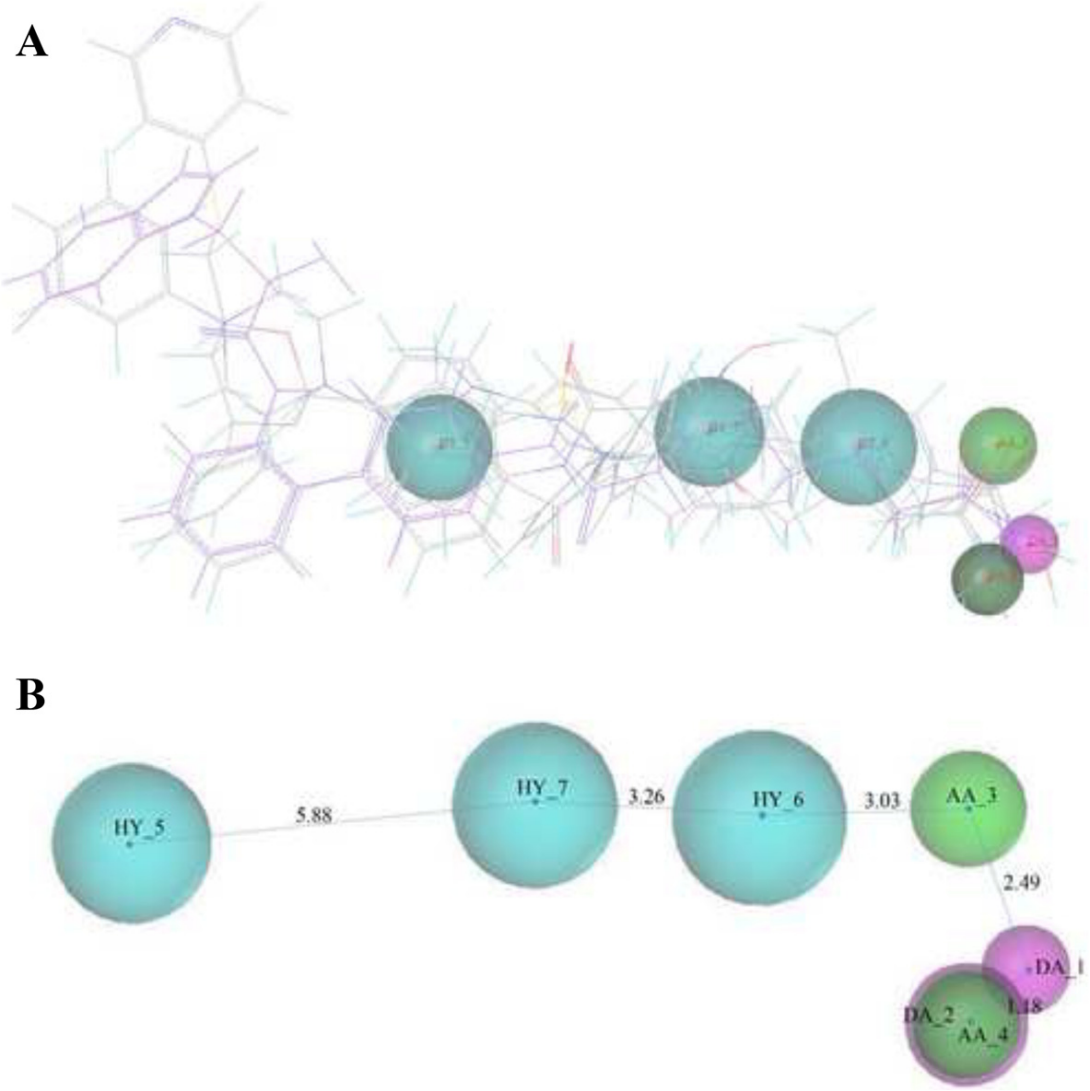
Pharmacophore MODEL_006 and its molecular alignment derived from the representative compounds. **a** Molecular alignment of 7 representative compounds. **b** Pharmacophore model (length unit: angstrom): three hydrophobes (HY5, HY6 and HY7), two hydrogen bond (HB) acceptors (AA3 and AA4), and two HB donors (DA1 and DA2). Cyan spheres represent hydrophobes; green spheres indicate HB acceptors; and magenta spheres indicate HB donors. Note that the pharmacophore AA_4 and DA_2 were overlapped each other

As listed in Table 3, the calculation results indicated that MODEL_006 had the best EF values. Moreover, MODEL_006 had the highest SPECIFICITY value, a moderate steric score, and an acceptable energy value. Therefore, it was selected as the final pharmacophore model. As shown in Fig. 1, MODEL_006 included seven pharmacophore features as follows: three hydrophobes (HY5, HY6 and HY7), two hydrogen bond (HB) acceptors (AA3 and AA4), and two HB donors (DA1 and DA2). Note that the pharmacophore AA_4 and DA_2 were overlapped each other. The hydrophobic moieties of the pharmacophore reflect the need for a hydrophobic region such as the linker domain or the cap group domain [33]. The HB acceptor and donor moieties of the pharmacophore reflect the need for the ZBG domain [27]. As a result, MODEL_006 was used as a 3D query to screen the Enamine database using the UNITY search module in SYBYL-X 2.0. In the seven features of MODEL_006, the maximum omitted features were set to two. The final 11,905 hits were retrieved.

**Table 3:** The EF values of the pharmacophore models for the test and decoy datebases

Name	Test dataset			Decoy dataset		
	Ht	Ha	EF	Ht	Ha	EF
MODEL_001	235	40	76.98723	8743	398	1.82089
MODEL_002	269	36	60.53086	9586	365	1.52305
MODEL_005	145	24	74.86345	8652	403	1.86315
MODEL_006	198	43	98.22677	8752	543	2.48172
MODEL_010	227	40	79.70044	7962	412	2.06983
MODEL_021	154	28	82.23636	8965	474	2.11489
MODEL_030	178	25	63.52528	9045	387	1.71144
MODEL_041	218	38	78.84128	9654	455	1.88523

### Molecular docking-based virtual screening

Initial validation of the docking protocol was performed by re-docking the ligand extracted from the HDAC 8 crystal structure (PDB id: 1 T64) to HDAC 8 itself. The top conformation of the ligand produced by GOLD 5.2 was very close to the crystal structure-bound conformation of the ligand. The root-mean-square deviation between the docked pose and its bound pose in the crystal structure was 0.75 Å. This indicated that GOLD 5.2 is able to reproduce the correct binding pose of an HDAC inhibitor ligand to its receptor. Next, the decoy dataset MUBD-HDACs [32] were further used to validate our docking protocol. 1 T64, 1 T67 and 1 T69 were used as the receptor protein and the four docking functions in GOLD 5.2 (ChemPLP, Goldscore, Chemscore and ASP) were used as scoring functions. The EF values and functional thresholds of ChemPLP, Goldscore, Chemscore and ASP for the top 1, 5, 10 and 20 % of ranked compounds in the decoy database were listed in Table 4. From the Table 4, we can see that the EF values of ChemPLP and Chemscore are obviously higher than that of Goldscore and ASP. The previous literature also pointed out that ChemPLP demonstrated the best results for both pose prediction and virtual screening [34]. So we chose ChemPLP and Chemscore together to further evaluate their screening results. As listed in Table 5, the combination of the two scoring functions is better in screening results than the two separate functions. Finally, ChemPLP was chosen as the main function and Chemscore was chosen as as the secondary scoring function for practical screening. After validation of the docking protocol, all 11,905 hits retrieved by pharmacophore model-based screening were docked into the active site of three crystal structures of HDAC8 (1 T64, 1 T67 and 1 T69). According to the actual situation, the about top 1 % of ranked compounds in the hit database were decided to remain for experimental validation. The score 72 for ChemPLP and 23 for ChemScore were selected as the final score thresholds. As a results, 154 compounds were selected as the hit compounds. Finally, the Selector module in SYBYL-X 2.0 was adopted for the clustering analysis by creating and comparing diverse subsets of the 154 hit compounds. 22 of the 154 hit compounds with diverse structures were selected as the final hits (listed in Table 6). We further examined the binding patterns of the 22 final hits (Additional file 1: Figure S1). As listed in Table 7, the binding patterns of the 22 hit compounds can be divided into five types. For the first class of compounds, their ZBG domain may form covalent metal chelate complexes and hydrogen bond interactions with HDAC residues. For the second class of compounds, their ZBG domain and the linker domain may form covalent metal chelate complexes and hydrogen bond interactions with HDAC residues. For the third class of compounds, its ZBG domain and cap group domain may form covalent metal chelate complexes and hydrogen bond interactions with HDAC residues GLY151 and TYR306. The fourth class of compounds included ZINC03260906 and ZINC09715944. The ZBG domain of ZINC03260906 may form covalent metal chelate complexes and its linker domain and the cap group domain may form hydrogen bond interactions with HDAC residues HIS180 and PHE208. The ZBG domain of ZINC09715944 may form covalent metal chelate complexes and its linker domain and the cap group domain may form hydrogen bond interactions with HDAC residues LYS33, HIS143, and SER150. The fifth class of compounds included ZINC02627831 and ZINC12581173. The linker domain of ZINC02627831 may form hydrogen bond interactions with HDAC residue ASP101. The ZBG domain of ZINC12581173 may form hydrogen bond interactions with HDAC residue LYS33.

**Table 4:** The EF values and functional thresholds for the top 1, 5, 10 and 20 % of the decoy database in individual docking function test

Docking functions	Top (%)	Functional thresholds	EF		
			1T64	1T67	1T69
ChemPLP	1	72.9	4.67	4.21	4.87
	5	68.3	4.47	4.15	4.68
	10	62.8	4.31	4.09	4.52
	20	56.9	4.14	4.02	4.44
Goldscore	1	60.1	1.87	1.37	1.14
	5	55.4	1.71	1.23	1.04
	10	50.1	1.68	1.19	0.98
	20	46.2	1.65	1.14	1.03
Chemscore	1	23.7	4.08	3.94	4.31
	5	19.1	3.98	3.81	4.18
	10	15.1	3.94	3.78	4.15
	20	12.7	3.87	3.72	4.06
ASP	1	35.3	1.45	1.21	1.78
	5	31.3	1.31	1.11	1.56
	10	28.4	1.29	1.03	1.38
	20	24.1	1.23	0.95	1.15

**Table 5:** The EF values and functional thresholds for the top 1, 5, 10 and 20 % of the decoy database in combined docking function test

Top (%)	Docking functions	EF			
	ChemPLP	Chemscore	1T64	1T67	1T69
1	71.8	22.8	4.84	4.54	4.98
5	67.4	17.6	4.37	4.21	4.43
10	62.1	14.2	3.53	3.84	3.81
20	57.5	12.3	3.24	3.31	3.42

**Table 6:** Structure of the 22 final compounds

cmpd.	ᅟ	cmpd.	ᅟ
ZINC01895726		ZINC12555961	
ZINC02627831		ZINC12581173	
ZINC02639234		ZINC23140995	
ZINC03260906		ZINC23141716	
ZINC03307410		ZINC23141899	
ZINC06178852		ZINC23143331	
ZINC06415107		ZINC23886004	
ZINC06497704		ZINC58161863	
ZINC09350495		ZINC60063267	
ZINC09715944		ZINC67907864	
ZINC11325463		ZINC84111476

**Table 7:** Different binding patterns of 22 hit compounds

Binding patterns	Hit names	Interaction types	Pharmacophore interaction regions
1	ZINC01895726, ZINC02639234, ZINC06178852, ZINC23140995, ZINC23141716, ZINC23143331, ZINC23886004, ZINC58161863, ZINC60063267, ZINC67907864, ZINC84111476	metal chelate bonds and hydrogen bonds	ZBG domain
2	ZINC03307410, ZINC06415107, ZINC06497704, ZINC09350495, ZINC12555961, ZINC23141899	metal chelate bonds and hydrogen bonds	ZBG domain and linker domain
3	ZINC11325463	metal chelate bonds and hydrogen bonds	ZBG domain and cap group domain
4	ZINC03260906, ZINC09715944	metal chelate bonds and hydrogen bonds	ZBG domain, linker domain and cap group domain
5	ZINC02627831, ZINC12581173	hydrogen bonds	ZBG domain or linker domain

### Inhibitory enzymatic activity evaluation

**Fig. 2:**
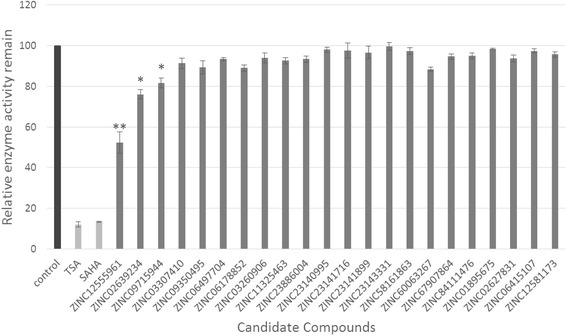
Inhibitory activity of the 22 hit compounds against HDACs. The enzymatic activities of the hit compounds are expressed as percentages of the positive control. Black bars represent the positive control, white bars represent the inhibitor control, and gray bars indicate the hit compounds being treated. Results are expressed as the mean ± SD (n ≥ 3). * mean *P* < 0.05 and ** mean *P* < 0.01

Based on the in silico results, additional *in vitro* studies were performed to evaluate the activity of the final 22 hit compounds. A fluorometric HDAC activity assay was firstly performed to examine the inhibitory activity of the 22 hit compounds against HDACs in HeLa nuclear extracts (Biovision K340-100). The experimental results are depicted in Fig. 2, which shows that three compounds, namely ZINC12555961, ZINC02639234, and ZINC09715944, inhibited the enzymatic activity of HDACs. Their relative enzymatic activities were 52 % (*P* = 0.008), 76 % (*P* = 0.006) and 82 % (*P* = 0.011), respectively, whereas that of the control inhibitor TSA was 12 % (*P* = 0.0003). The other 19 hit compounds did not show significant inhibitory activity against HDACs.

**Fig. 3:**
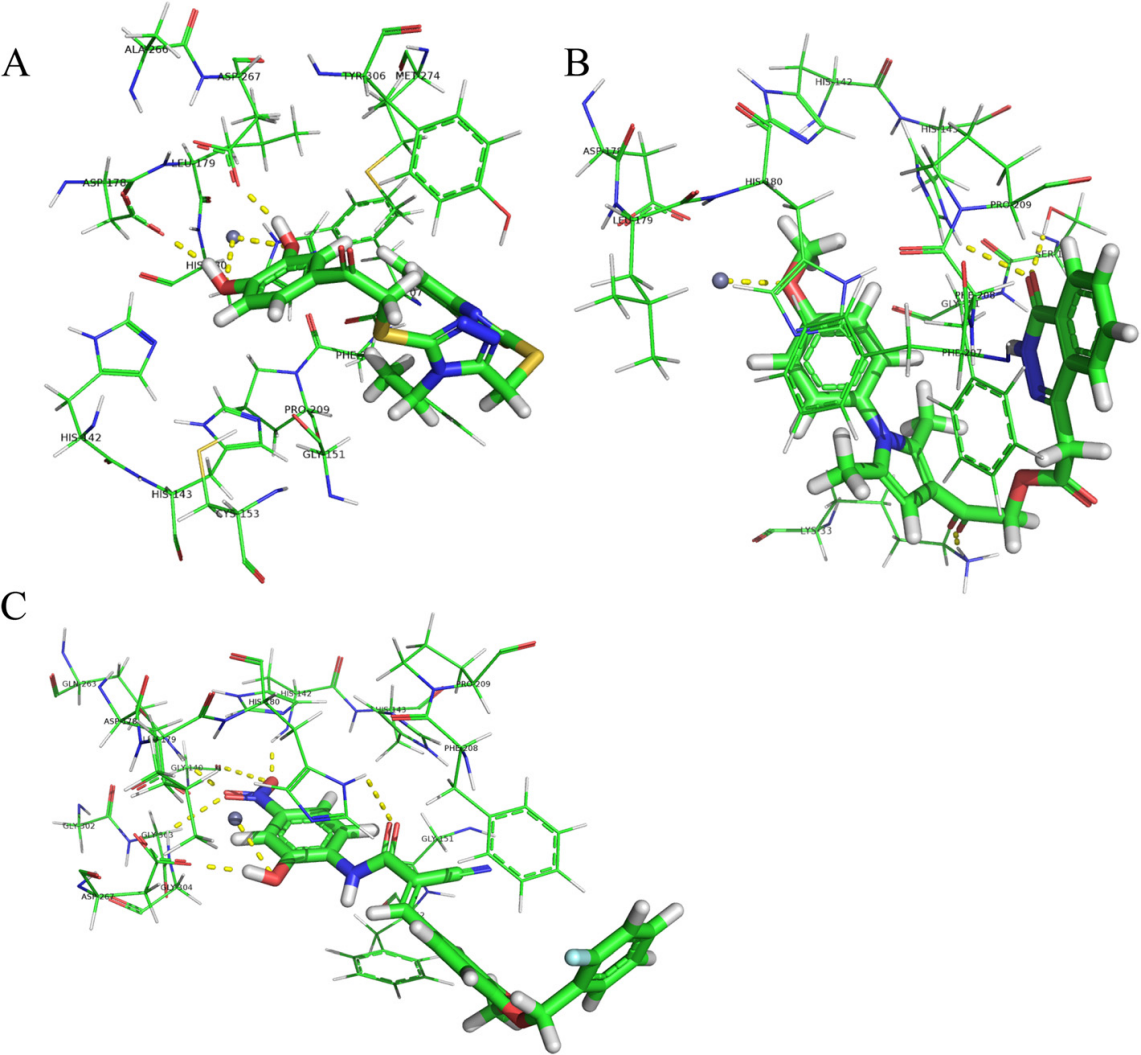
Molecular docking results. Docked orientations of **a** ZINC02639234, **b** ZINC09715944, and **c** ZINC12555961. Active site residues are shown by lines and the metal ion (Zn^2+^) is shown by a grey sphere. The hydrogen bond network with protein residues and the metal ion is represented by a yellow dotted line

The three active compounds did not belong to either of the four main classes of HDAC inhibitors, namely hydroxamic acids, aliphatic acids, benzamides, and cyclic peptides [9–11]. ZINC12555961 has four main functional groups: fluorophenyl, methoxyphenyl, acrylamide cyanide and nitrophenyl; ZINC02639234 has three main functional groups: benzothiazole, triazole and dihydroxy phenyl; and ZINC09715944 has three main functional groups: benzene and pyridazine ketone, pyrrole and methoxypheny. The docking poses of the three active compounds to the active site of HDAC8 (1 T69) are shown in Fig. 3. The two hydroxyls of the dihydroxy phenyl group of ZINC02639234 formed covalent chelate complexes with zinc ions and hydrogen bond interactions with HDAC residues ASP178 and ASP267. In addition, the benzothiazole group of ZINC02639234 had hydrophobic contact with HDAC residue PHE208 (Fig. 3a). For ZINC09715944, its methoxyphenyl group formed covalent chelate complexes with zinc ions, the carbonyl of its linker domain formed hydrogen bond interactions with HDAC residue LYS33, and the carbonyl of its benzene and pyridazine ketone group was not only able to form hydrogen bond interactions with HDAC residues HIS143 and SER150, but was also able to form hydrophobic contact with HDAC residue PHE208 (Fig. 3b). For ZINC12555961, the hydroxyl of its nitrophenyl group was not only able to form covalent chelate complexes with zinc ions, but was also able to form hydrogen bond interactions with HDAC residue ASP267; the nitro of its nitrophenyl group could form hydrogen bond interactions with HDAC residues GLY304, GLN263, GLY140 and HIS142; the carbonyl group of its acrylamide cyanide was able to form hydrogen bond interactions with HDAC residue HIS180, and its methoxyphenyl group was in hydrophobic contact with HDAC residue PHE208 (Fig. 3c). The three compounds were selected for further cytotoxicity assays.

### Anti-proliferative activity and apoptosis-inducing mechanism

**Fig. 4:**
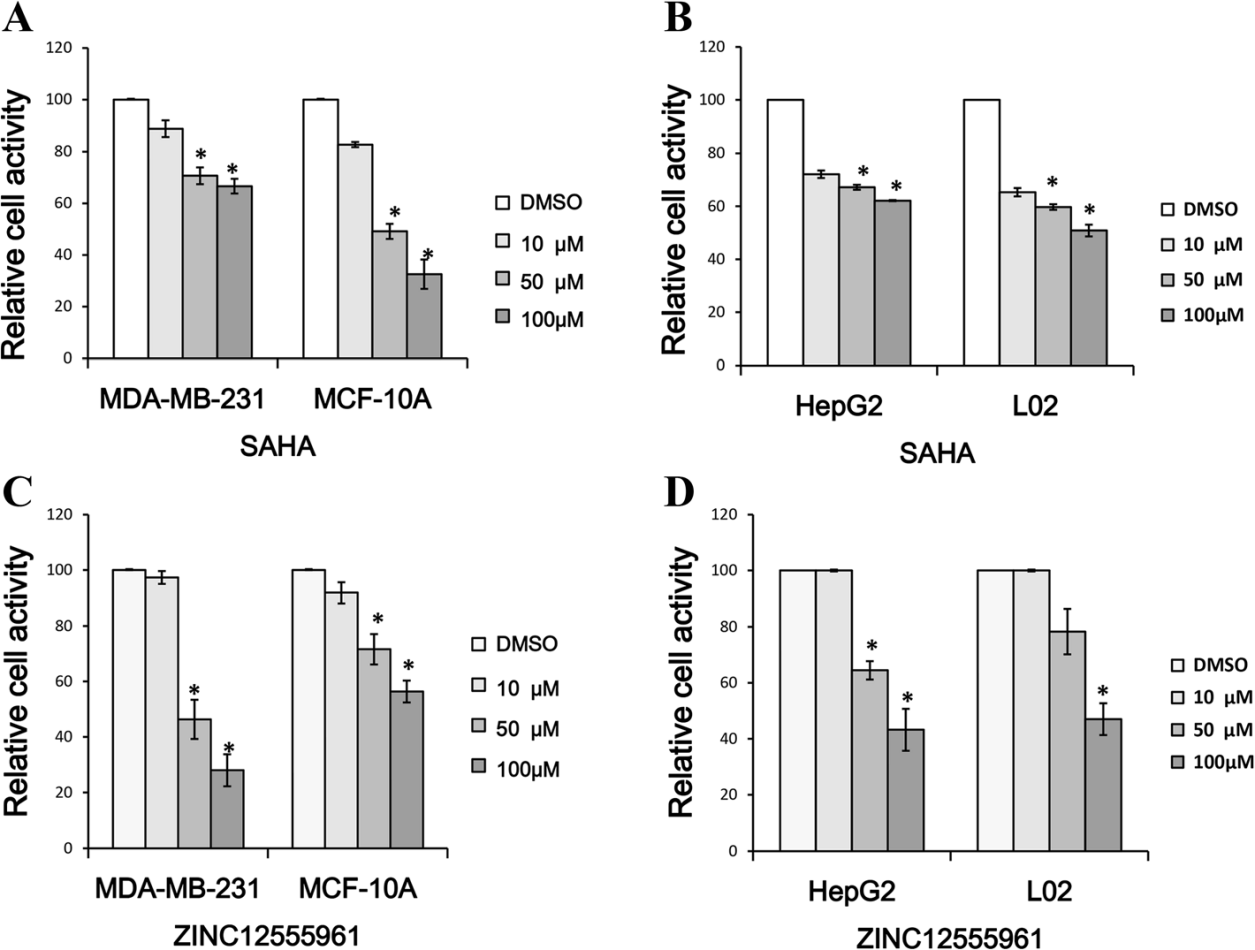
Comparison of the cytotoxicity of ZINC12555961 and SAHA against cancer cells and normal cells. **a** MDA-MB-231 and MCF-10A cells were treated with 0, 10, 50 and 100 μM SAHA. **b** HepG2 and L02 cells were treated with 0, 10, 50 and 100 μM SAHA. **c** MDA-MB-231 and MCF-10A cells were treated with 0, 10, 50 and 100 μM ZINC12555961. **d** HepG2 and L02 cells were treated with 0, 10, 50 and 100 μM ZINC12555961. Significance was determined by the Student’s *t*-test. The values represent as the mean ± S.D. * means *P* < 0.05

HDAC inhibitors selectively induce cell growth arrest and apoptosis in a wide variety of cancer cells. To test the cytotoxicity of the three hit compounds with HDAC inhibitory activities, MTT, DAPI staining, and Annexin V-FITC assays were designed and performed according to the procedures described in the Materials and methods section. In the MTT assay, HepG2, L02, MDA-MB-231, and MCF-10A cells were cultured in 3 % serum-supplemented medium and treated with four different concentrations (1, 10, 50, and 100 μM) of the three hit compounds and SAHA. The viabilities of the four cell lines after 48 h of treatment were measured using the MTT assay. The IC50 values for ZINC12555961, ZINC02639234, ZINC09715944 and SAHA against the four cell lines were calculated and are listed in Table 8, which shows that ZINC02639234 and ZINC09715944 exhibited stronger toxicity towards normal cells than against cancer cells, whereas ZINC12555961 showed stronger toxicity towards cancer cells than normal cells. Increasing ZINC12555961 concentrations (from 0 to 100 μM) led to a steady decrease in the viability of MDA-MB-231cells (IC50 = 57 μM), but had a less toxic effect on MCF-10A cells (IC50 = 142 μM). In the HepG2 and L02 cell lines, ZINC12555961 exhibited nearly the same toxicity, with IC50 values of 87 and 85 μM, respectively. Furthermore, in our experiments, ZINC12555961 was more potent than SAHA in inhibiting the viability of cancer cells (Fig. 4). The IC50 values of SAHA in HepG2, L02, MDA-MB-231, and MCF-10A cells were 166 μM, 85 μM, 178 μM, and 59 μM, respectively. The results indicated that ZINC12555961 has inhibitory activity against all four cells and displays promising and selective inhibitory activity against cancer cell viability, ZINC09715944 has inhibitory activity against HepG2, L02, and MCF-10A cells but not against MDA-MB-231 cell and does not display selective inhibitory activity against cancer cell viability, and zinc02639234 has no inhibitory activity against all four cells.

**Table 8:** Comparison of the IC50 values of SAHA, ZINC12555961, ZINC02639234 and ZINC09715944 against the HepG2, L02, MDA-MB-231 and MCF-10A cell lines

Chemicals	IC50(μM)			
	HepG2	L02	MDA-MB-231	MCF-10A
SAHA	166 ± 9	85 ± 2	178 ± 13	59 ± 10
ZINC12555961	87 ± 10	85 ± 15	57 ± 7	142 ± 17
ZINC02639234	>200	>200	>200	>200
ZINC09715944	157 ± 12	65 ± 3	>200	47 ± 7

Data are expressed as the mean ± SD from at least three independent experiments

**Fig. 5:**
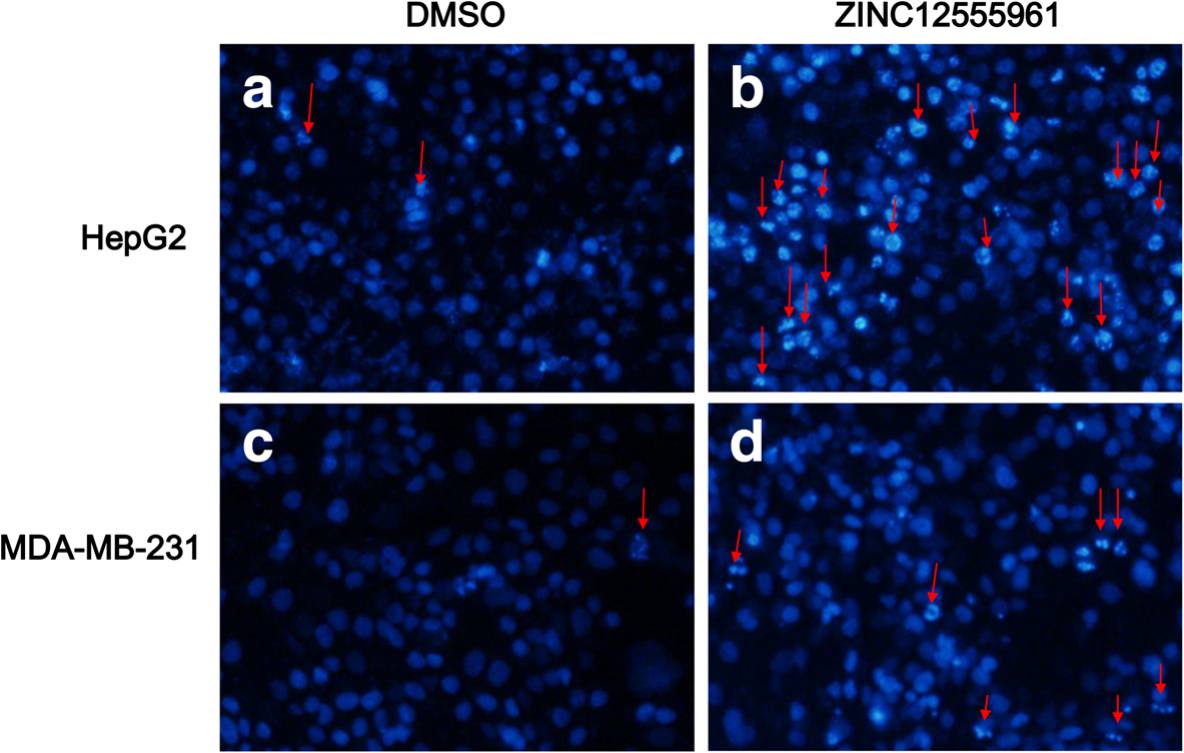
Nuclear morphological changes and apoptotic HepG2 and MDA-MB-231 cells treated with ZINC12555961 (90 μM) and DMSO for 48 h. Arrows indicate apoptotic nuclei. **a** HepG2 cells by DAPI staining treated with DMSO. **b** HepG2 cells by DAPI staining treated with ZINC12555961. **c** MDA-MB-231 cells by DAPI staining treated with DMSO. **d** MDA-MB-231 cells by DAPI staining treated with ZINC12555961

As ZINC12555961 could remarkably suppress the viability of HepG2 and MDA-MB-231 cells, a DAPI staining assay was performed to determine whether its inhibitory effect on cell viability is associated with the induction of cell apoptosis. Fluorescent microscopic images of DAPI stained nuclei of HepG2 and MDA-MB-231 cells treated with ZINC12555961 or DMSO for 48 h are shown in Fig. 5. The concentrations of zinc12555961 used in HepG2 and MD-231 cells were 90 μM and 60 μM, respectively. Apoptotic nuclei in both cell lines were split into several nuclear apoptotic bodies, and apoptotic cells are shown in deep white by DAPI staining, as indicated by the red arrows (Fig. 5b and d), whereas cells treated with DMSO exhibited round intact nuclei. These results indicate that ZINC12555961 may play an important role in inducing cancer cell apoptosis.

**Fig. 6:**
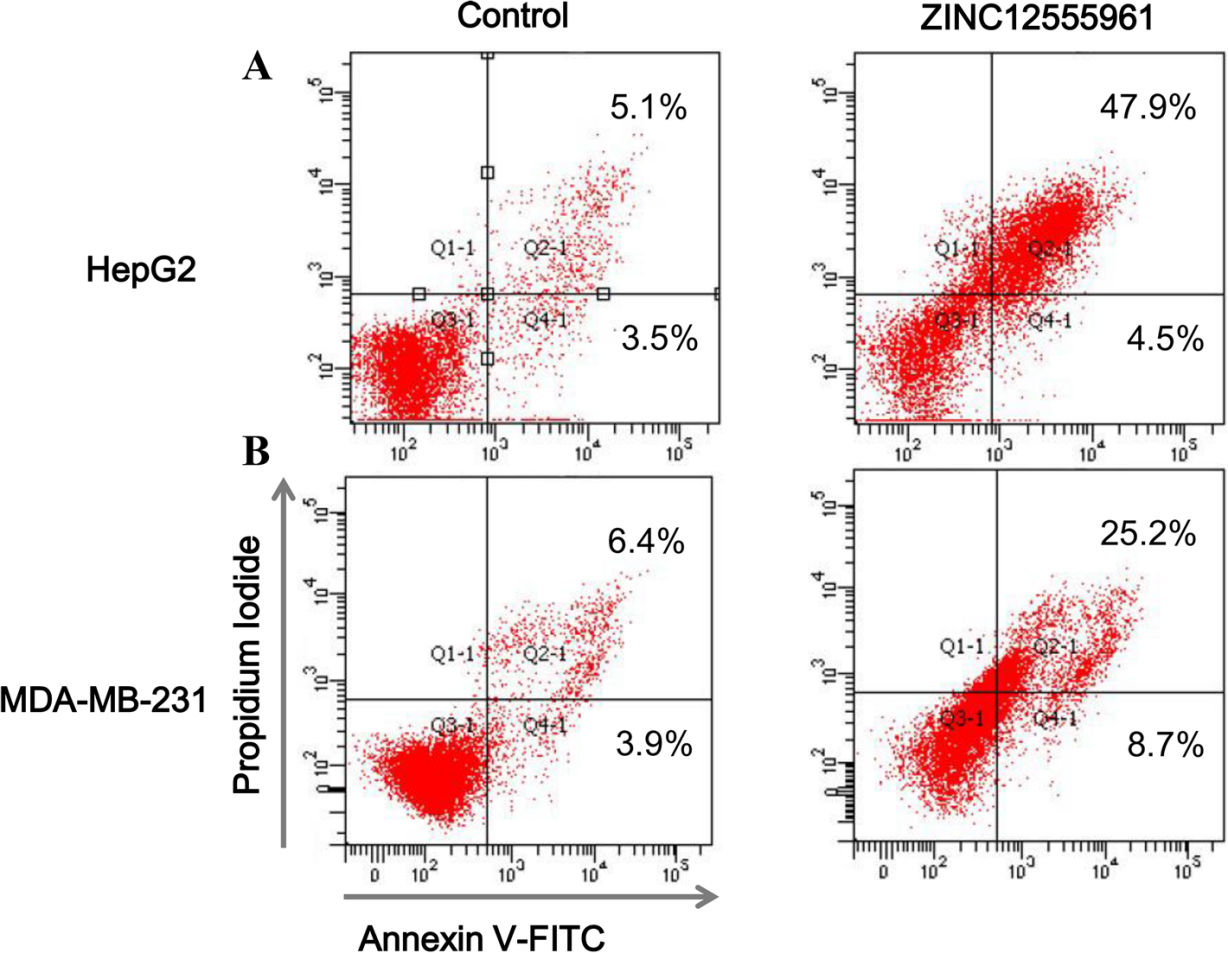
Quantitative analysis of the effects of ZINC12555961 on the apoptosis of human cancer cell lines. HepG2 cells **a** and MDA-MB-231 cells **b** were treated with DMSO or 90 μM ZINC12555961 for 48 h. Cells were harvested by trypsinization and centrifugation, stained with Annexin V-FITC and PI, and analyzed by flow cytometry. Representative results are shown

Flow cytometric analysis with Annexin V-FITC conjugated to PI was performed to further examine the effect of ZINC12555961 on cancer cell apoptosis. HepG2 and MDA-MB-231 cells were treated with DMSO (90 μM) or ZINC12555961 (90 μM) for 48 h. Apoptotic cells were stained and monitored by flow cytometry (Fig. 6). In both HepG2 and MDA-MB-231 cell lines, ZINC12555961 significantly induced the late apoptotic stage. The apoptosis rates of HepG2 cells treated with DMSO and ZINC12555961 were 5.1 and 47.9 %, respectively, and those of MDA-MB-231 cells treated with DMSO and ZINC12555961 were 6.4 and 25.2 %, respectively. ZINC12555961 also affected the early apoptotic stage in both cell lines. The early apoptosis rates of the control group vs. the experimental group in HepG2 and MDA-MB-231 cells were 3.5 % vs. 4.5 % and 3.9 % vs. 8.7 %, respectively. These results indicate that the rates of apoptosis induced by ZINC12555961 were significantly higher than those of the control groups. ZINC12555961 may play an important role in inducing cancer cell apoptosis.

**Fig. 7:**
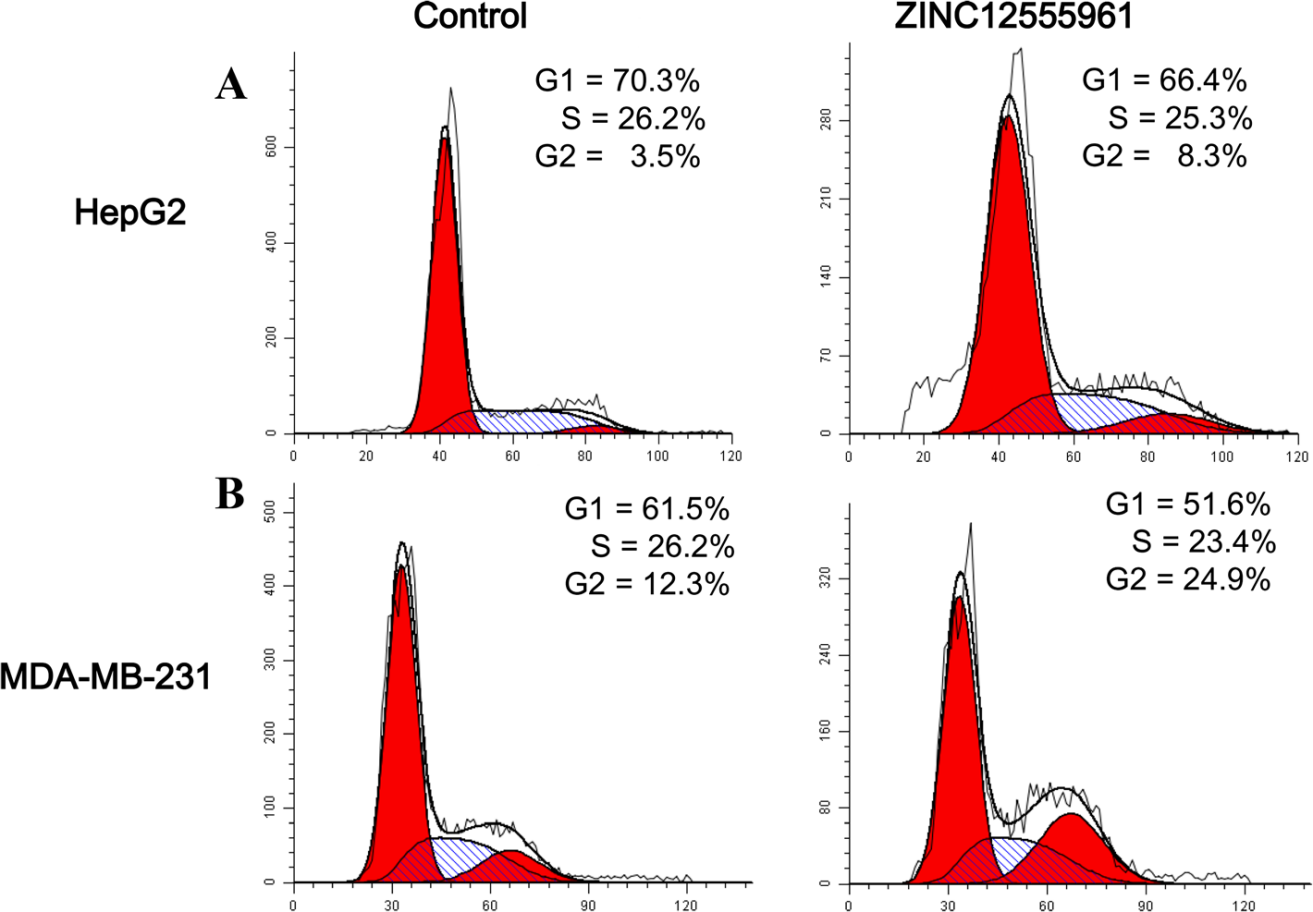
Effects of ZINC12555961 on cell cycle progression in cancer cells. HepG2 cells **a** and MDA-MB-231 cells **b** were treated with DMSO or ZINC12555961 for 32 and 48 h. At the end of treatment, cells were trypsinized, incubated with RNase, stained with PI, and analyzed by flow cytometry. Representative results are shown

As shown above, the effects of ZINC12555961 on apoptosis, as measured by flow cytometry, do not explain the decrease of cell viability measured by MTT assay. This suggests that ZINC12555961 may affect cell viability through other mechanisms. Therefore, we tested the effect of ZINC12555961 on cell cycle distribution by flow cytometric analysis. HepG2 and MDA-MB-231 cells were treated with ZINC12555961 for 32 and 48 h and analyzed by flow cytometry (Fig. 7). In the HepG2 cell line, the percentage of cells in the G_1_ phase decreased from 70.3 to 66.4 % in response to ZINC12555961, and this decrease was accompanied by an increase in the proportion of cells in the G_2_ phase from 3.5 to 8.3 % (Fig. 7a). Similarly, in MDA-MB-231 cells, the percentage of cells in the G_1_ phase decreased from 61.5 to 51.6 % in response to ZINC12555961, and this decrease was accompanied by an increase in the proportion of cells in the G_2_ phase from 12.3 to 24.9 % (Fig. 7b). This indicates that the inhibitory effect of ZINC12555961 on the proliferation of HepG2 and MDA-MB-231 cells may be associated with G_2_/M phase cell cycle arrest.

## Discussions

HDAC enzymes have emerged as exciting and promising novel targets for the treatment of cancer, diabetes and other human diseases. HDAC inhibitors, as a new class of potential therapeutic agents, have attracted a great deal of interest both for research and clinical applications. Computer aided drug design (CADD) and virtual screening have been applied in the development of new HDAC inhibitors. Many HDAC inhibitors were designed and synthesized based on CADD approaches [35–41]. Certain potent HDAC inhibitors with novel structures were identified by virtual screening approaches [31, 42, 43]. Vadivelan et al. developed a pharmacophore model based on common chemical features of HDAC inhibitors [44]. Melagrakia et al. developed a linear five-parameter quantitative structure-activity relationship (QSAR) model of HDAC inhibitors [45]. Xiang et al. developed a pharmacophore model and three QSAR models for a series of benzimidazole and imidazole inhibitors of HDAC2 [46]. Zhao et al. used a two-step modeling approach to study the selectivity and activity of HDAC inhibitors [47]. Thangapandian et al. used pharmacophore modeling and molecular docking approaches for the identification of potential HDAC8 inhibitors [48]. More recently, Thangapandian et al. used a combined pharmacophore modeling, molecular docking and molecular dynamics (MD) simulation approach for the identification of potential HDAC8 inhibitors [49]. Nair et al. used a combined pharmacophore modeling, flexible docking, and three-dimensional QSAR (3D–QSAR) approach for the identification of benzimidazole and imidazole derivatives [50]. Although these studies did not experimentally validate the activities of their candidate compounds, their use of virtual screening approaches for HDAC inhibitors provides support for further computational and experimental research. Park et al. identified novel classes of HDAC inhibitors with new zinc-chelating groups using docking simulations, and experimentally validated the activities of their candidate compounds [42]. Tang et al. identified three hit compounds using a combinatorial QSAR screening model based on support vector machine and k-Nearest Neighbors algorithms, and experimentally confirmed the inhibitory activities of the compounds against HDAC1 [31]. Zhang et al. identified a potent HDAC inhibitor with a novel scaffold using ZBG (zinc-binding group)-based virtual screening, and experimentally confirmed the inhibitory activities of the compounds against HDAC8 [43]. In the present study, we developed a hierarchical virtual screening protocol for the identification of potential HDAC inhibitor compounds. The multistage virtual screening workflow was used to screen and identify 22 final hit compounds, and the HDAC inhibitory activities of three of the 22 compounds, namely ZINC12555961, ZINC02639234 and ZINC09715944, were experimentally validated by *in vitro* enzyme inhibition assays. The results confirmed the efficacy and validity of our screening method. The three active compounds showed a novel structure that does not belong to the previously reported four classes of HDAC inhibitors. All three active hits showed different scaffolds, thereby providing wide opportunities for future HDAC inhibitor design. The novelty of the 22 final hit compounds was assessed using SciFinder scholar (https://scifinder.cas.org/). The SciFinder results confirmed that these compounds were not previously tested for HDAC inhibitory activity.

We further examined the cytotoxicity of the three hit compounds with HDAC inhibitory activities against the human normal liver cell line, L02, and the liver cancer cell line, HepG2, as well as the human breast cancer cell line, MDA-MB-231, and the human breast epithelial cell line, MCF-10A. The MTT assay results demonstrated that the active compound ZINC12555961 could selectively suppress the viability of human cancer cell lines (HepG2 and MDA-MB-231 cells). Staining with DAPI and Annexin V-FITC/PI flow cytometry assays revealed that the effect of ZINC12555961 on cancer cell death may be mediated by the induction of apoptosis and G_2_/M phase cell cycle arrest. These results indicate that ZINC12555961 is a promising HDAC inhibitor and has anti-tumor potential. Future studies will be aimed at elucidating the molecular mechanisms underlying ZINC12555961-induced selective cancer cell apoptosis and evaluating the isoform-selective HDAC inhibitory effects of ZINC12555961. ZINC12555961-focused virtual screening will also be further developed in the future.

## Conclusions

In conclusion, the study identified three new HDAC inhibitors. The new-found HDAC inhibitors are worthy to further investigations.

## Abbreviations

AA, Hydrogen bond acceptors; CADD, Computer aided drug design; DMSO, Dimethyl sulfoxide; EF, Enrichment factor; ELISA, Enzyme-linked immunosorbnent assay; FITC, Fluorescin isothiocyanate; GA, Genetic algorithm; HB, Hydrogen bond donors; HB, Hydrogen bond; HDAC, Histone deacetylases; HepG2, Human hepatocellular carcinoma cell line; HY, Hydrophobes; L02, Human normal liver cell line; MCF-10A, Human normal breast cell line; MDA-MB-231, Human breast cancer cell line; MTT, 3-(4, 5-dimethylthia-zol-2-yl)-2, 5-diphenyl tetrazolium bromide; PBS, Phosphate-buffered saline; PDB, Protein data bank; PI, Propidium iodide; SAHA, Suberoylanilide hydroxamic acid; TSA, Trichostatin A; ZBG, Zinc-binding group

## Additional file

Additional file 1: Figure S1. Comparisons of 22 ligand’s binding poses against 1T69. (DOCX 8808 kb)
