# Supplementary material for: Virtual screening and experimental validation of novel histone deacetylase inhibitors
Source: BMC Pharmacol Toxicol. 2016 Jul 21;17:32. doi: 10.1186/s40360-016-0075-8 (PMC4955146; doi:10.1186/s40360-016-0075-8)
Supplement: Additional file 1: Figure S1. — Comparisons of 22 ligand’s binding poses against 1T69. (DOCX 8808 kb) [file 40360_2016_75_MOESM1_ESM.docx]

**Fig. S1. Comparisons of 22 ligand’s binding poses against 1T69.** Atoms are colored by their types. Protein residues are represented by lines and ligands are represented by sticks. Active site residues are shown in lines and metal ion (Zn^2+^) is shown in grey sphere. Hydrogen bond interactions between ligands and protein residues (including metal ion) are represented in dotted yellow lines.

**
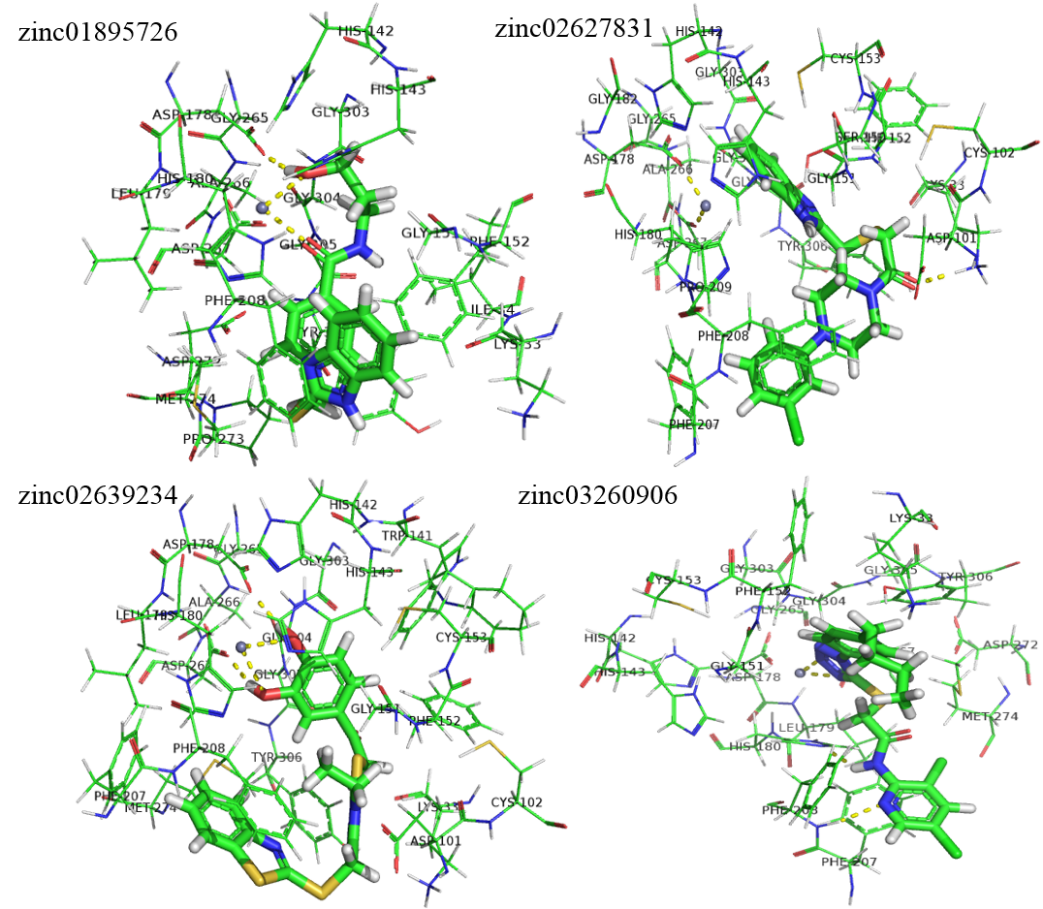

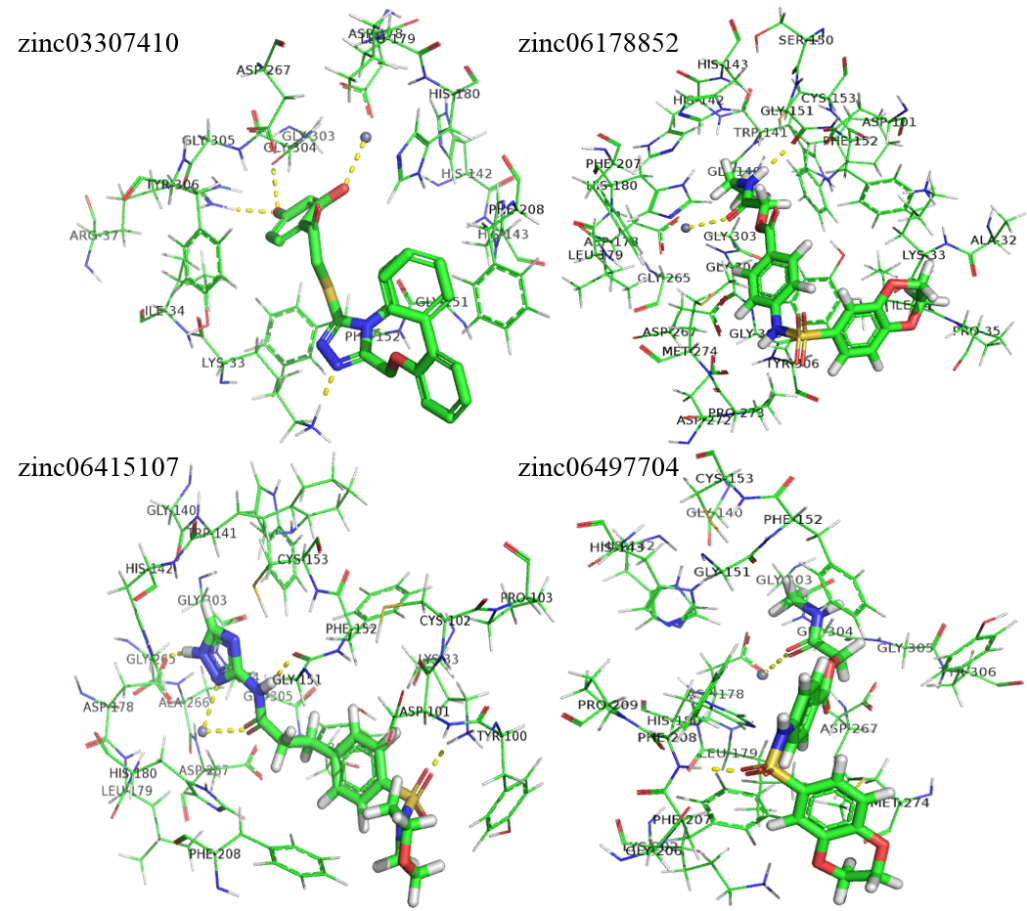
**

**Figure S1.** Cont.

**
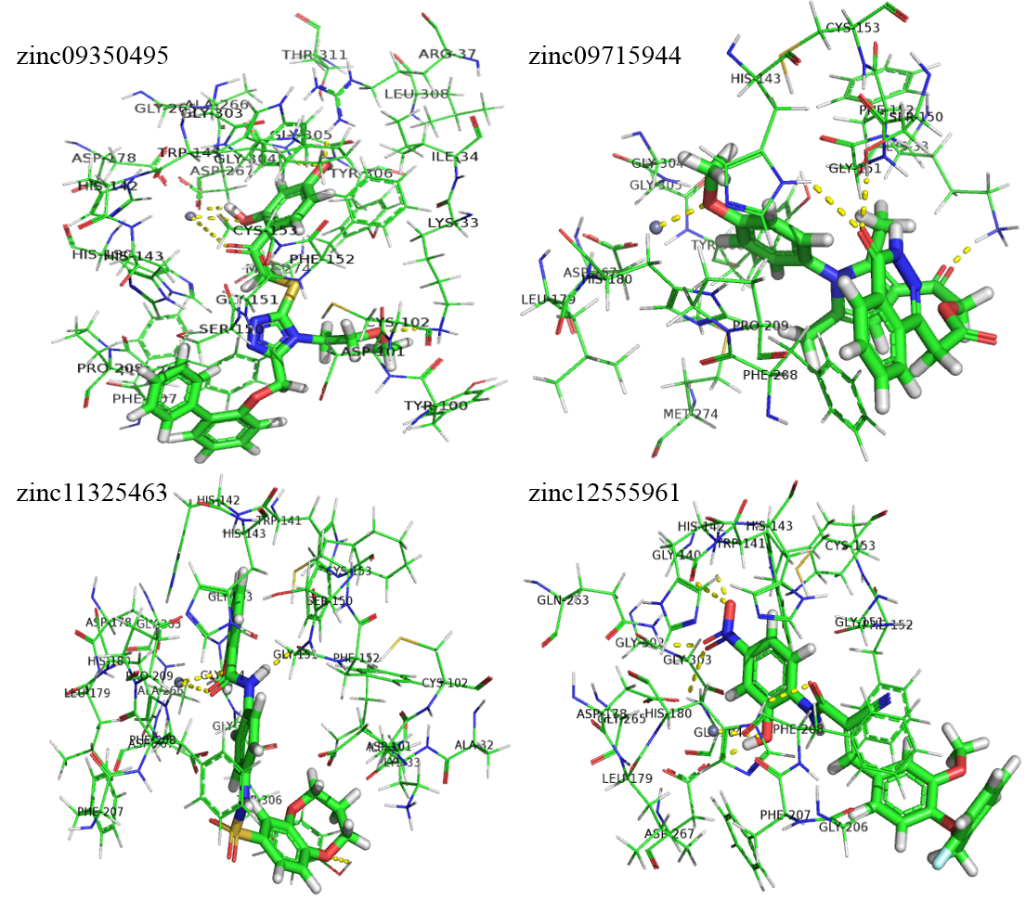

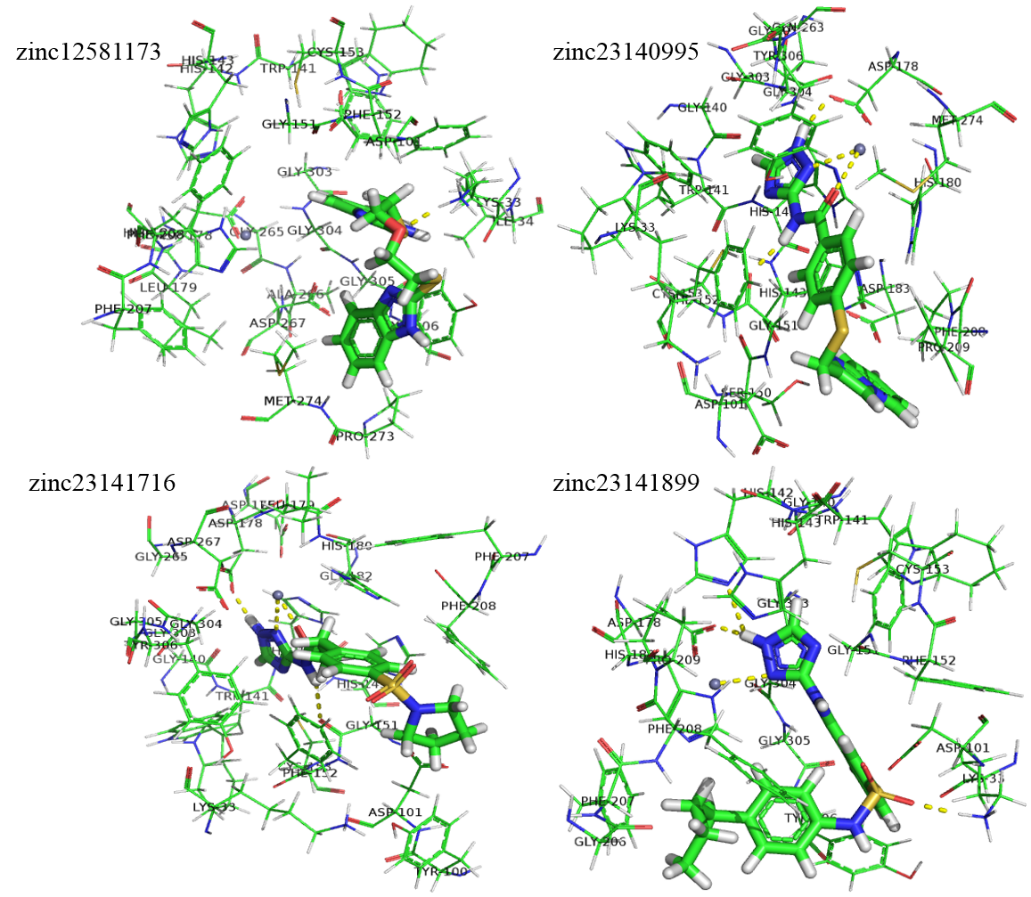
**

**Figure S1.** Cont.

**
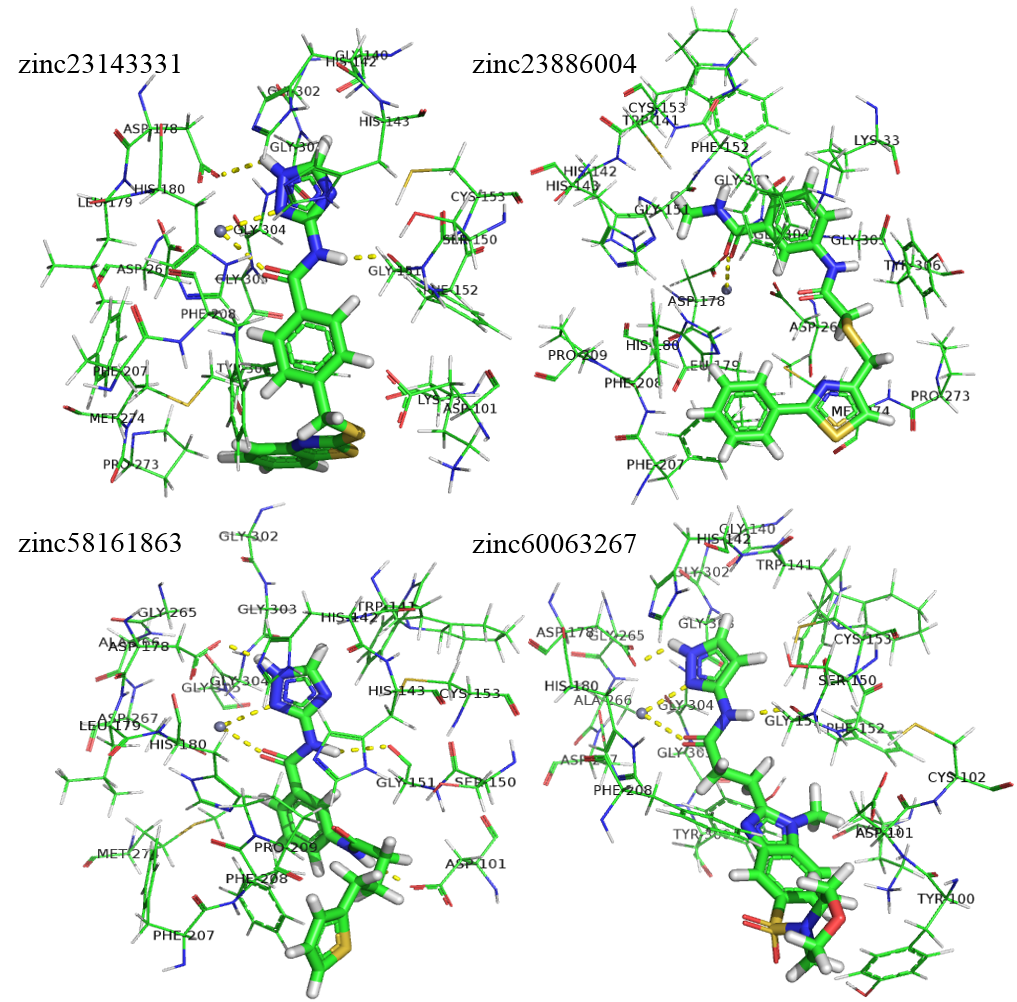

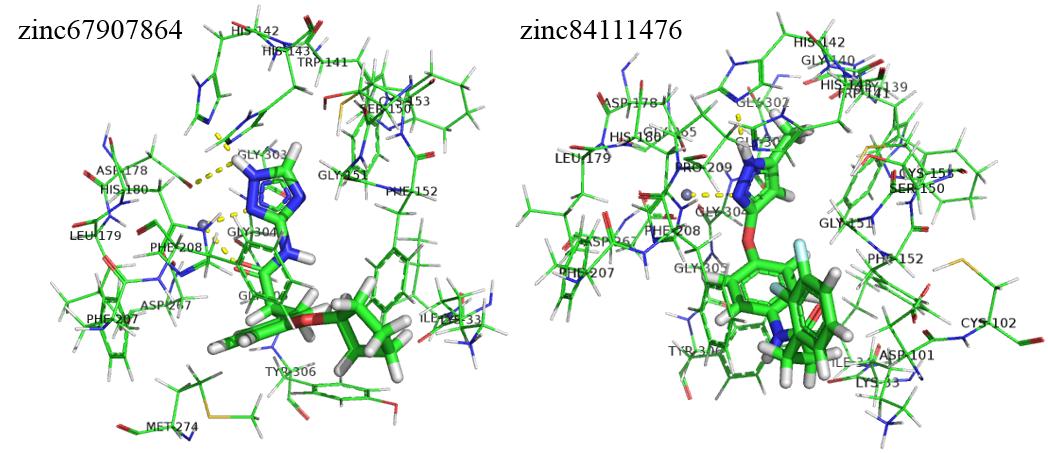
**
